# Five Silkworm 30K Proteins Are Involved in the Cellular Immunity against Fungi

**DOI:** 10.3390/insects12020107

**Published:** 2021-01-27

**Authors:** Lin Ye, Yan Zhang, Zhaoming Dong, Pengchao Guo, Dongchao Zhao, Haoyun Li, Hang Hu, Xiaofang Zhou, Haiqin Chen, Ping Zhao

**Affiliations:** 1 State Key Laboratory of Silkworm Genome Biology, Southwest University, Chongqing 400716, China; yelin3968@wchscu.cn (L.Y.); zhangy66@swu.edu.cn (Y.Z.); dongzhaoming@swu.edu.cn (Z.D.); guopc@swu.edu.cn (P.G.); zdczdc@swu.edu.cn (D.Z.); lhy_3014777@163.com (H.L.); 2 Biological Science Research Center, Southwest University, Chongqing 400716, China; 3 Department of Biotechnology, College of Biotechnology, Southwest University, Chongqing 400716, China; huhang19970@163.com (H.H.); lqq39178694@163.com (X.Z.); chq1245@126.com (H.C.)

**Keywords:** *Bombyx mori*, 30K proteins, fungi, cellular immunity

## Abstract

**Simple Summary:**

The molecular mechanism of 30K proteins in anti-fungal immunity remains unclear. Here, we examined the mRNA levels of 30K proteins, including BmLP1, BmLP2, BmLP3, BmLP4, and BmLP7, and found that all of these proteins were significantly upregulated after injection of pathogen-associated molecular patterns to the fifth instar larvae, implying their involvement in immune response. The binding assay results showed that only BmLP1 and BmLP4 can bind to both fungal cells and silkworm hemocytes. In vitro, the encapsulation of hemocytes on day 5 of the fifth instar larval stage was promoted by the coating of agarose beads with recombinant BmLP1 and BmLP4. Therefore, these results demonstrate that 30K proteins are involved in the cellular immunity of silkworms by acting as pattern recognition molecules to directly recruit hemocytes to the fungal surface. We believe that our study makes a significant contribution to the literature because it provides insights into the 30K-mediated cellular immunity in silkworms.

**Abstract:**

Background: 30K proteins are a major group of nutrient storage proteins in the silkworm hemolymph. Previous studies have shown that 30K proteins are involved in the anti-fungal immunity; however, the molecular mechanism involved in this immunity remains unclear. Methods: We investigated the transcriptional expression of five 30K proteins, including BmLP1, BmLP2, BmLP3, BmLP4, and BmLP7. The five recombinant 30K proteins were expressed in an *Escherichia coli* expression system, and used for binding assays with fungal cells and hemocytes. Results: The transcriptional expression showed that the five 30K proteins were significantly upregulated after injection of pathogen-associated molecular patterns to the fifth instar larvae, indicating the possibility of their involvement in immune response. The binding assay showed that only BmLP1 and BmLP4 can bind to both fungal cells and silkworm hemocytes. Furthermore, we found that BmLP1-coated and BmLP4-coated agarose beads promote encapsulation of hemocytes in vitro. The hemocyte encapsulation was blocked when the BmLP1-coated beads were preincubated with BmLP1 specific polyclonal antibodies. Conclusions: These results demonstrate that 30K proteins are involved in the cellular immunity of silkworms by acting as pattern recognition molecules to directly recruit hemocytes to the fungal surface.

## 1. Introduction

Insect hemolymph is the circulatory fluid that surrounds all organs and contributes to physiological processes [1]. The components of hemolymph, such as free amino acids, sugars, lipids, phosphorylcholine, and proteins, have been previously investigated [2,3,4]. Hemolymph proteins are synthesized in the fat body and secreted into the hemolymph [5,6], where they perform important functions, including transporting nutrients, wastes, CO_2_, and hormones. They have also been reported to be involved in the innate immune response [7,8,9,10].

In the hemolymph of silkworms (*Bombyx mori*, an important model of insect), abundant nutrient storage and immunity-related proteins have been identified. The nutrient storage proteins include apolipophorins, storage proteins, and 30K proteins, and the immunity-related proteins include immune recognition, signaling, and effectors [11]. The 30K proteins are a major group of nutrient storage proteins in the silkworm hemolymph. A total of forty-six 30K proteins have been identified, all of which belong to the lipoprotein 11 family, and in 1978, were defined as low molecular weight lipoproteins (LPs) [6,12].

In our previous study, we divided the 30K proteins into the following three subfamilies: typical 30K proteins (BmLP1–BmLP24), serine/threonine-rich 30K proteins (BmLP25–BmLP36) and ENF peptide-binding proteins (BmLP37–BmLP46) [12]. ENF peptides have various biological activities, including growth blocking and paralysis. The first three amino acid residues are E, N, and F, which are highly conserved [13]. Among them, the typical 30K proteins are synthesized in the fat body and secreted into the hemolymph during the fifth instar larval and early pupal stages [14,15]. The crystal structure of three typical 30K proteins has been determined. All these proteins contain two domains, namely, an all-*α* N-terminal domain (NTD) and an all-*β* C-terminal domain (CTD) [16,17,18]. The NTD had a putative lipid-binding cavity, whereas the CTD was similar to the carbohydrate-binding domain, the ricin B-type domain of mosquitocidal holotoxin (with two galactose-binding sites) [19].

Carbohydrate-binding proteins play critical roles in activating the immune system by recognizing carbohydrates on the surfaces of pathogens [20]. A previous study has shown that the BmLP1 can bind to the *β*-1,3 glucan [21], and interfere with the hyphal growth of the entomopathogenic fungus *Paecilomyces tenuipes* in the pupae of the silkworm [22]. 30K proteins have been considered to be involved in the immune response of silkworm, but their detailed roles and mechanisms are still unclear.

In the present study, we investigated the expression patterns of five 30K proteins in the silkworm after being induced by *β*-1,3 glucan and mannan. The five 30K proteins were expressed in an *Escherichia coli* expression system, and were used to analyze the binding ability with fungal cells and hemocytes in the *Candida albicans*-silkworm system. Although *C. albicans* is not a natural insect fungal pathogen, the *C. albicans*-silkworm system has been often used to study fungal infections of insects, since *C. albicans* is a pathogenic and unicellular fungus, and calculating the cell number of injections is, therefore, fairly straightforward [23,24]. Furthermore, our study clarified the defense mechanism of 30K proteins against fungal infection.

## 2. Materials and Methods

### 2.1. Sample Preparation

The *Bombyx mori* strain of Dazao was maintained in the State Key Laboratory of Silkworm Genome Biology at the Southwest University of China. The larvae were reared on fresh mulberry leaves at room temperature, 75% ± 5% relative humidity, and a photoperiod of 12 h light/12 h dark. The fat body of fifth instar larvae on day 3 was frozen in liquid nitrogen, and then stored at −80 °C for the extraction of total RNA. The fungus *C. albicans* (Bei Chuang Biological, China) was stored in potato liquid medium at 4 °C.

### 2.2. Bioinformatics Analysis

The amino acid sequences of BmLP1 (NCBI (National Center for Biotechnology Information): NM_001044021.2), BmLP2 (NCBI: XM_012695032.2), BmLP3 (NCBI: XM_012695050.2), BmLP4 (NCBI: XM_012695085.2), and BmLP7 (NCBI: XM_012694938.2) were downloaded from NCBI, for multiple sequence alignments of the five 30K proteins of the silkworm. Alignments were performed using ClustalW [25] and ESPript [26].

### 2.3. Immune Injection in Silkworm

The pathogen-associated molecular pattern (PAMP) molecules of *β*-1,3 glucan and mannan (Shanghai yuanye Bio-Technology, Shanghai, China) were dissolved in phosphate-buffered saline (PBS) (20 mM Na_2_HPO_4_, 20 mM NaH_2_PO_4_ and 50 mM NaCl, pH 7.4), with final concentrations of 1.0 and 0.5mg/mL, respectively. Day 1 fifth instar larvae were randomly divided into three groups, each containing more than 30 individuals. The injections were carried out through the stomata on the surface of silkworm larvae at a dose of 10 μL β-1,3 glucan, mannan, or PBS, the latter being used as a control. After injection, five individuals from each group were randomly collected at 8, 16, 24, 32, 40, and 48 h, respectively. The fat body was collected for the RT-qPCR experiments, and the hemolymph was collected for western blotting.

### 2.4. Total RNA Isolation and cDNA Synthesis

The fat body from different time points of fifth instar larvae was used to extract the total RNA. The samples were first homogenized, and then the total RNA was isolated using TRIzol reagent (Invitrogen, Carlsbad, CA, USA). Reverse transcription into cDNA was performed using M-MLV reverse transcriptase (Invitrogen, Carlsbad, CA., USA) with oligo (dT)-adaptor as the primer. The reaction mixtures were incubated at 75 °C for 5 min, and then heated at 42 °C for 1 h. The concentration of cDNA was measured by spectrophotometry at 260 nm. The cDNA mixture was diluted to 200 ng/μL and stored at −20 °C.

### 2.5. Real-Time Quantitative Polymerase Chain Reaction

The expression patterns of *Bmlp1*, *Bmlp2*, *Bmlp3*, *Bmlp4* and *Bmlp7* after stimulation with PAMPs were assayed by real-time quantitative polymerase chain reaction (RT-qPCR). The RT-qPCR was performed on the qTOWER2.2 qPCR System (Analytik Jena Biometra, Jena, Germany) using SYBR Premix Ex Taq (TaKaRa, Kyoto, Japan). The PCR amplifications were performed in triplicate. The gene for silkworm eukaryotic translation initiation factor 4A (SilkDB Probe: sw22934) was used as the reference gene. The relative expression content was calculated using the relative quantitative method (2^−ΔΔCt^), and the Student’s *t*-test was used to calculate statistical significance.

### 2.6. Expression and Purification of the Recombinant Protein

The coding regions of *Bmlp1* (NCBI: NM_001044021.2), *Bmlp2* (NCBI: XM_012695032.2), *Bmlp3* (NCBI: XM_012695050.2), *Bmlp4* (NCBI: XM_012695085.2), and *Bmlp7* (NCBI: XM_012694938.2) were amplified using the Primer Star 2× Mix (TaKaRa, Kyoto, Japan) from the fat body cDNA of day 3 fifth instar larvae, using a specific primer (Table S1). The PCR products were then separated by 1% agarose gel electrophoresis, purified using the PCR Clean-Up System (Promega, Madison, WI., USA), and ligated to the expression vector pNIC-28 Bsa4 at 22 °C. The recombinant proteins were expressed in an *E. coli* system BL21(DE3) (TransGen Biotech, Beijing, China) and purified by Ni-NTA Agarose affinity chromatography and Superdex 75 separation chromatography (GE Healthcare, Chicago, IL., USA). The concentration of purified proteins was measured using the BCA Protein Assay Kit (Beyotime, Shanghai, China). The proteins were stored at −80°C until further use.

### 2.7. Binding Assay of Recombinant 30K Proteins

*C. albicans* was cultured in a potato liquid medium for 20 h at 30 °C. It was then collected by centrifugation at 1500 rpm and washed three times with PBS (20 mM Na_2_HPO_4_/NaH_2_PO_4_, and 50 mM NaCl; pH 7.5). The precipitate was suspended in phosphate-buffered saline (PBS). Recombinant BmLP1, BmLP2, BmLP3, BmLP4, and BmLP7 were added to a final concentration of 0.1 mg/mL. Samples containing no proteins were used as the controls. The solutions were rotated for 2 h at room temperature to ensure complete binding. After incubation, the cells were collected by centrifugation at 1500 rpm and washed four times with PBS. Further, 5× SDS-PAGE loading buffer was added to the washed *C. albicans* cells. The sample was incubated in boiling water for 5 min. Finally, SDS-PAGE and western blotting were performed for the binding assay.

Hemolymph was collected from day 5 fifth instar larvae and mixed with an equal volume of Grace’s cell culture medium (10% fetal bovine serum (FBS), 0.1% penicillin–streptomycin, and 200 μL of 10mM phenylthiourea). The diluted hemolymph was added to each well of a 12-well cell plate (Corning, Corning, NY., USA) and hemocytes were allowed to adhere to the slide for 10 min at room temperature. The plasma was then removed and the hemocytes were washed three times with PBS. Recombinant BmLP1 was added to final concentrations of 0.1, 0.2, and 0.4 mg/mL; the sample containing no proteins was used as the control. The cell plate was incubated at 27 °C for 2 h, and then the medium was removed. The hemocytes were washed three times with PBS and extracted using RIPA buffer (Beyotime, Shanghai, China), and the concentration of cell extracts was measured using the BCA Protein Assay kit (Beyotime, Shanghai, China). The hemolymph protein samples were separated by SDS-PAGE.

### 2.8. Sodium Dodecyl Sulfate Polyacrylamide Gel Electrophoresis and Western Blotting

The 8 μg hemolymph protein samples were separated by SDS-PAGE (12% (*w*/*v*) poly- acrylamide gel). For the western blotting assay, the proteins were transferred onto a PVDF (polyvinylidene fluoride) membrane after separation by SDS-PAGE, and blocked with 5% skimmed milk at room temperature for 2 h. Mouse anti 6× his-tag monoclonal antibodies (1:20,000) were added and incubated at room temperature for 1 h. The membrane was washed three times with TBST (Tris Buffered saline Tween), each for 10 min. The HRP (Horseradish Peroxidase)-conjugated goat anti-mouse IgG (1:20,000) was added and incubated at room temperature for 1 h, and then washed four times with TBST, each for 10 min. SuperSignal^®^ West Pico Chemiluminescent Substrate (Thermo Fisher Scientific, Waltham, MA, USA) was used for visualization.

### 2.9. Immunofluorescence

Hemolymph was collected from day 5 fifth instar larvae and mixed with equal volumes of Grace’s cell culture medium (10% FBS, 0.1% penicillin–streptomycin and 200 μL 10 mM phenylthiourea). The diluted hemolymph was added to each well of a 24-well cell plate (Corning, Corning, NY, USA) with a round coverslip, and the hemocytes were allowed to adhere to the slide for at least 10 min at room temperature. Subsequently, the plasma was removed and the hemocytes were washed three times with PBS. Fresh medium with recombinant BmLP1, BmLP2, BmLP3, BmLP4, and BmLP7 (final concentration of 0.4 mg/mL) was added, and the sample without proteins was used as the control. After incubation at 27 °C for 2 h, the medium was removed and the hemocytes were washed three times with PBS. The cells were fixed in 4% formaldehyde at room temperature for 10 min, permeated with 0.3% Triton X-100 for 15 min, and blocked with 1% bovine serum albumin and 10% normal goat serum solution for 2 h at room temperature. The cells were then incubated with mouse anti 6× his-tag antibodies (1:500) overnight at 4 °C, and washed three times with PBS. Alexa Fluor 488-labeled goat anti-mouse IgG (1:500) was used to visualize the proteins, and DAPI (4′,6-diamidino-2-phenylindole) was used to stain the nucleus. The slides were examined under a fluorescence microscope.

### 2.10. Non-Reduce PAGE

The recombinant BmLP1, BmLP2, BmLP3, BmLP4, and BmLP7 were incubated with different concentrations of SDS (0.5%, 1%, and 2%) for 20 min. The non-reduced sample loading buffer (5×) without βME (2-Hydroxy-1-ethanethiol) was added to the samples. The polymers and monomer were isolated using non-reduced PAGE and stained using Coomassie Brilliant Blue R250 (Sangon Biotect, Shanghai, China).

### 2.11. In Vitro Encapsulation

Ni-NTA agarose beads (Qiagen, Dusseldorf, Germany) were equilibrated in TBS (Tris-HCl pH 8.0, 100mM NaCl); recombinant His-tagged BmLP1, BmLP2, BmLP3, BmLP4, and BmLP7 were added and incubated with the agarose beads with shaking at 4 °C overnight. The coated beads were then washed three times with TBS.

A 48-well cell culture plate was treated with 1% agarose. Hemolymph was collected from day 5 fifth instar larvae and mixed with an equal volume of Grace’s cell culture medium (10% FBS, 0.1% penicillin-streptomycin, and 200 μL 10 mM phenylthiourea). Diluted hemolymph was added to each well and the hemocytes were allowed to settle down for at least 10 min at room temperature. Subsequently, 1 μL of BmLP1-coated, BmLP2-coated, BmLP3-coated, BmLP4-coated, and BmLP7-coated agarose beads were added to each well, and beads without protein coatings were used as controls. The plate was incubated at 27 °C, and the agarose beads were observed after 6 h of incubation. For each recombinant protein, the assay was performed in two different wells.

To test whether in vitro encapsulation can be blocked by BmLP1 specific polyclonal antibodies, 10 μL BmLP1-coated beads were incubated with 20 μL BmLP1 specific polyclonal antibodies with shaking at 4 °C overnight. The beads were then washed with PBS for three times and resuspended in 10 μL PBS. An in vitro encapsulation assay was performed in the same manner as described above. The assay was performed in three different wells for statistical analysis.

## 3. Results

### 3.1. Sequence Analysis of Five 30K Proteins

**Figure 1:**
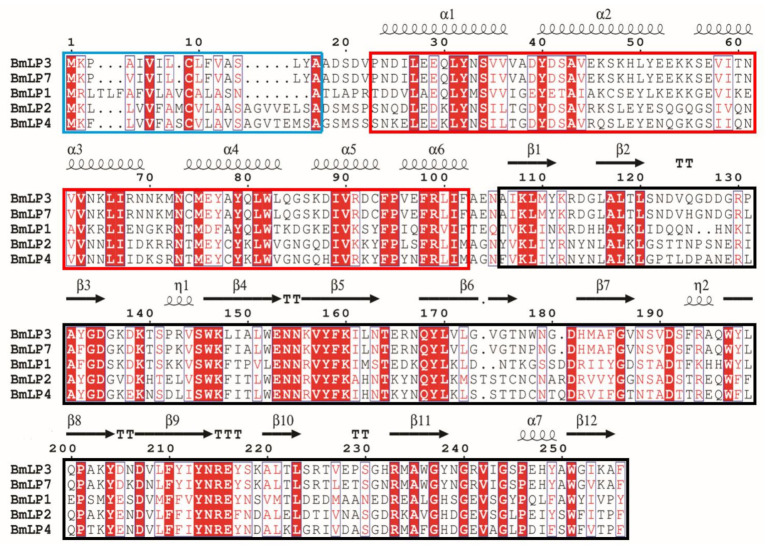
Multiple sequence alignments of five 30K proteins of silkworm. Alignments were Alignments were performed using ClustalW and ESPript. The second structure elements are indicated above the primary sequences, and the conserved residues are shaded in red, and the signal peptides are marked by blue border. The lipid-binding domains are marked by red border. The ricin domains are marked by black border.

Sequence analysis determined that the five typical 30K proteins, BmLP1, BmLP2, BmLP3, BmLP4, and BmLP7, have signal peptides composed of 17–23 amino acid residues (Figure 1), indicating that they could be secreted into the extracellular milieu. After removal of the signal peptide, the matured protein was predicted to be 27.5–27.9 kDa. BLAST analysis showed that mature 30K proteins consist of an all-α N-terminal domain (α1–α6, ~90 amino acids) and an all-β C-terminal domain (β1–β12, ~150 amino acids) (Figure 1). Multiple sequence alignment returned that the five typical 30K proteins shared 24–26% identity (Figure 1).

### 3.2. PAMPs Upregulate the Expression of 30K Genes

**Figure 2:**
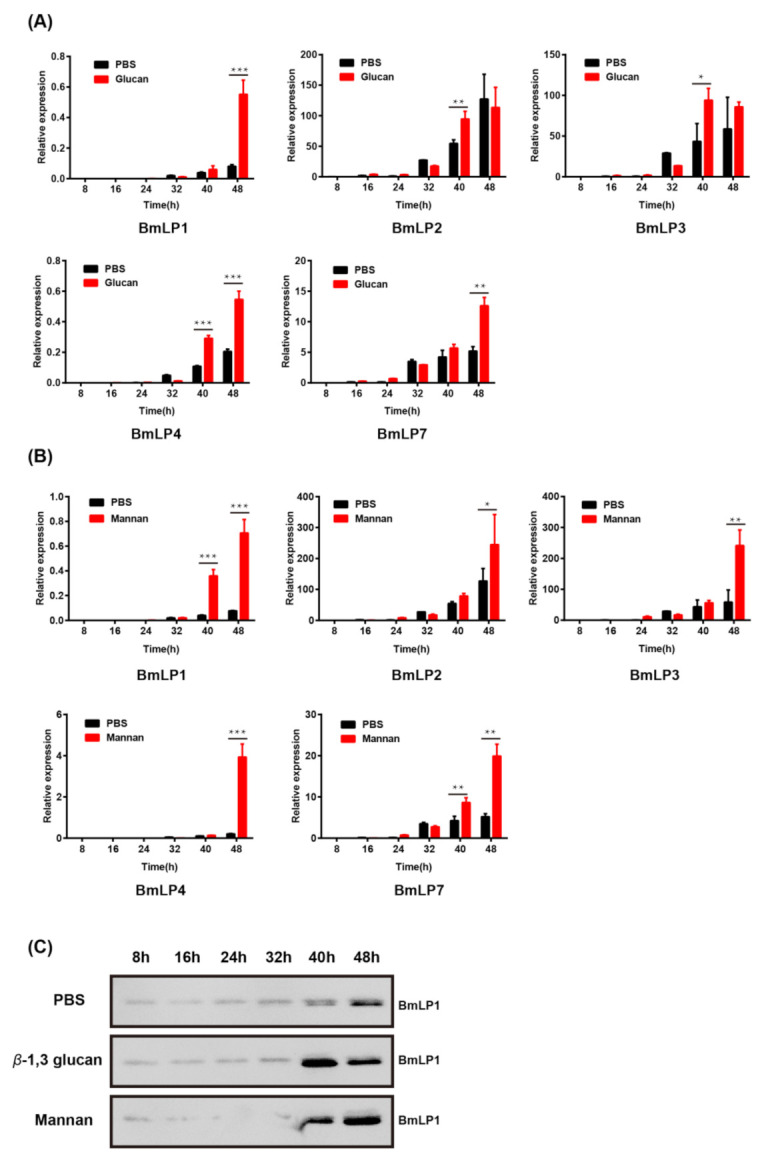
The mRNA expression of *Bmlp1*, *Bmlp2*, *Bmlp3*, *Bmlp4* and *Bmlp7* in the fat body after *β*-1,3 glucan-injected (**A**) and mannan-injected (**B**) at different time points was assayed using qPCR. The y-axis indicates the relative expression level of mRNA transcripts. The vertical bars represent the mean ± SE (n = 3). Student’s *t*-test was used to calculate statistical significance. *** *p* < 0.001, ** *p* < 0.01, * *p* < 0.1 versus the control. The error bars indicate the standard error of the mean (n = 3). The protein level of BmLP1 in the hemolymph of *β*-1,3 glucan, mannan, and phosphate-buffered saline (PBS)-injected larvae at different time points was detected by western blotting (**C**).

*β*-1,3 glucan and mannan are PAMPs from fungal cell walls, and these two PAMPs can stimulate a complex immune response [27]. To evaluate whether 30K genes’ expression can be upregulated by injection with the two PAMPs, the RT-qPCR was performed to determine the mRNA expression of *Bmlp1*, *Bmlp2*, *Bmlp3*, *Bmlp4*, and *Bmlp7* after *β*-1,3 glucan and mannan injection (Figure 2A,B) using specific primers (Table S1). In the *β*-1,3 glucan- injected group, the mRNA expression of *Bmlp1* and *Bmlp4* transcripts was significantly (*p* < 0.01) upregulated at 48 h compared with that of the PBS-injected group (Figure 2A). Similarly, the *Bmlp1* and *Bmlp4* transcripts were also found to be significantly upregulated (*p* < 0.01) at 40 and 48 h in the mannan-injected group (Figure 2B). The mRNA expression of *Bmlp2*, *Bmlp3*, and *Bmlp7* was also upregulated after *β*-1,3 glucan and mannan injection (Figure 2A,B). BmLP1 specific polyclonal antibodies were used to detect BmLP1 in the hemolymph after injection by western blotting. There were clear bands in the *β*-1,3 glucan- and mannan-injected groups at 40 and 48 h compared with those in the PBS-injected group (Figure 2C). The results showed that BmLP1 expression was significantly upregulated in the *β*-1,3 glucan-, and mannan-injected groups, which was consistent with the results of the mRNA levels.

### 3.3. Expression and Purification of Recombinant 30K Proteins

**Figure 3:**
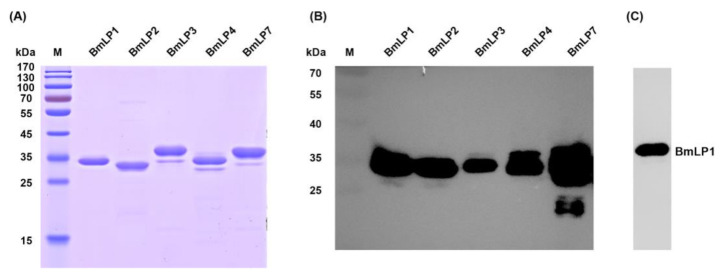
The purified recombinant proteins were detected by SDS-PAGE and western blotting. The SDS-PAGE gel was stained with Coomassie Brilliant Blue R250 (**A**) and western blotting was performed using mouse anti 6× his-tag antibodies and HRP (Horseradish Peroxidase)-conjugated goat anti mouse antibodies (**B**). The purified recombinant BmLP1 was detected by western blotting with BmLP1 specific polyclonal antibodies (**C**).

To verify whether 30K proteins can bind to fungus, we attempted to express recombinant BmLP1, BmLP2, BmLP3, BmLP4, and BmLP7 using the expression vector pNIC-28 Bsa4 and *E. coli* (BL21). Sodium dodecyl sulfate polyacrylamide gel electrophoresis (Figure 3A) and western blotting (Figure 3B) were performed to analyze the purified recombinant 30K proteins. The results showed that BmLP1, BmLP2, BmLP3, BmLP4, and BmLP7 have molecular masses of approximately 30 kDa. High-purity proteins were obtained after purification by Ni-NTA agarose affinity chromatography (Supplementary Figure S1). All recombinant 30K proteins were recognized by mouse anti-his-tag antibodies (Figure 3B), and BmLP1 can be recognized by BmLP1 specific polyclonal antibodies (Figure 3C).

### 3.4. 30K Proteins Can Bind to Fungus

**Figure 4:**
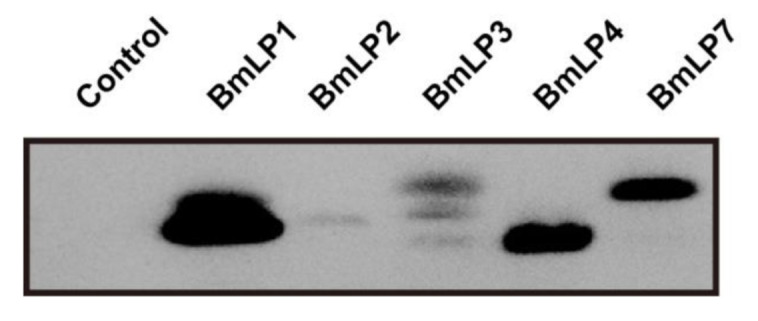
Fungal binding assay of BmLP1, BmLP2, BmLP3, BmLP4 and BmLP7 revealed using western blotting. Purified proteins in PBS were incubated with fungal under shaking for 2 h at room temperature. Subsequently, the fungal cells were pelleted, washed four times with PBS, and resuspended in PBS. Western blotting was performed using mouse anti 6× his-tag antibodies and HRP-conjugated goat anti mouse antibodies. Control: no protein.

The recognition of foreign pathogens is the first necessary step in invertebrate immune defense. As BmLP1 is known to be a fungal *β*-1,3 glucan-binding protein, we examined the binding ability of recombinant 30K proteins to fungi. No signal was detected in the control, which contained no protein, whereas a clear band was observed for the sample incubated with BmLP1, BmLP4, and BmLP7. However, the bands that were incubated with BmLP2 and BmLP3 were weak. These results revealed that the binding ability of BmLP1, BmLP4, and BmLP7 to fungi was stronger than that of BmLP2 and BmLP3 (Figure 4).

### 3.5. 30K Proteins Can Bind to Hemocytes

**Figure 5:**
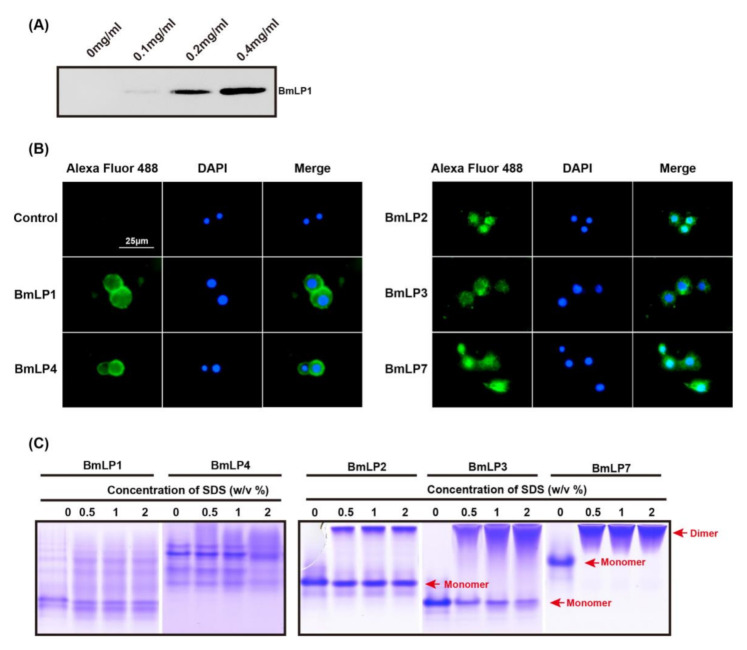
Binding of BmLP1 to hemocytes of the fifth instar larvae revealed using western blotting (**A**). Different concentrations of purified proteins in Grace’s medium were incubated with hemocytes for 2 h at room temperature, washed four times with PBS, and hemocyte proteins were extracted using RIPA Lysis Buffer. BmLP1 was detected using mouse anti 6× his-tag antibodies and HRP-conjugated goat anti mouse antibodies. The sample with no protein was used as the control. The binding and location of BmLP1, BmLP2, BmLP3, BmLP4 and BmLP7 in hemocytes were revealed by immunofluorescence (**B**). Purified proteins in Grace’s medium were incubated with hemocytes for 2 h at room temperature and washed four times with PBS. The proteins were detected using mouse anti 6× his-tag antibodies and Alexa Fluor 488-labeled Goat anti mouse antibodies. The sample with no protein was used as the control. The dimerization of BmLP1, BmLP2, BmLP3, BmLP4 and BmLP7 were performed by non-reduce PAGE (**C**). The recombinant BmLP1 and BmLP3 were incubated with different concentrations of SDS for 20 min. The final concentrations of SDS were 0.5%, 1% and 2%. The dimers were isolated using non-reduce PAGE and stained using Coomassie Brilliant Blue R250.

It has been shown that C-type lectins can bind to hemocytes in *Drosophila*. As the CTD domain of the 30K protein is a lectin-like domain, we investigated whether BmLP1 can also bind to hemocytes. Hemocytes were incubated with different concentrations of recombinant BmLP1 (0, 0.1, 0.2, and 0.4 mg/mL) for 2 h at room temperature. After incubation, hemocytes were washed three times with PBS and lysed with RIPA buffer. The binding assay of recombinant BmLP1 with hemocytes was performed by western blotting (Figure 5A). There was no signal in lane 1, in which the sample contained no protein, whereas BmLP1 was detected with a weak band in the lane with the sample containing 0.1 mg/mL protein. Clear bands were observed in the lanes with samples containing 0.2 and 0.4 mg/mL protein. The results showed that recombinant BmLP1 is able to bind to hemocytes. To investigate the location of 30K proteins in hemocytes, the hemocytes were either incubated with recombinant BmLP1, BmLP2, BmLP3, BmLP4, or BmLP7 (0.4 mg/mL), or without protein (control) and immunofluorescence was performed (Figure 5B). The BmLP1 and BmLP4 signals were observed on the surface of hemocytes, whereas the BmLP2, BmLP3, and BmLP7 signals were indicated in the cytoplasm of hemocytes; there was no fluorescence signal in the control group. The results showed that recombinant BmLP1 and BmLP4 can bind to the hemocyte membrane, whereas recombinant BmLP2, BmLP3, and BmLP7 can penetrate the cytoplasm of hemocytes. BmLP2, BmLP3, and BmLP7 could dimerize, but BmLP1 and BmLP4 were not able to dimerize in the study (Figure 5C).

### 3.6. BmLP1 and BmLP4 Promotes Hemocyte Encapsulation

**Figure 6:**
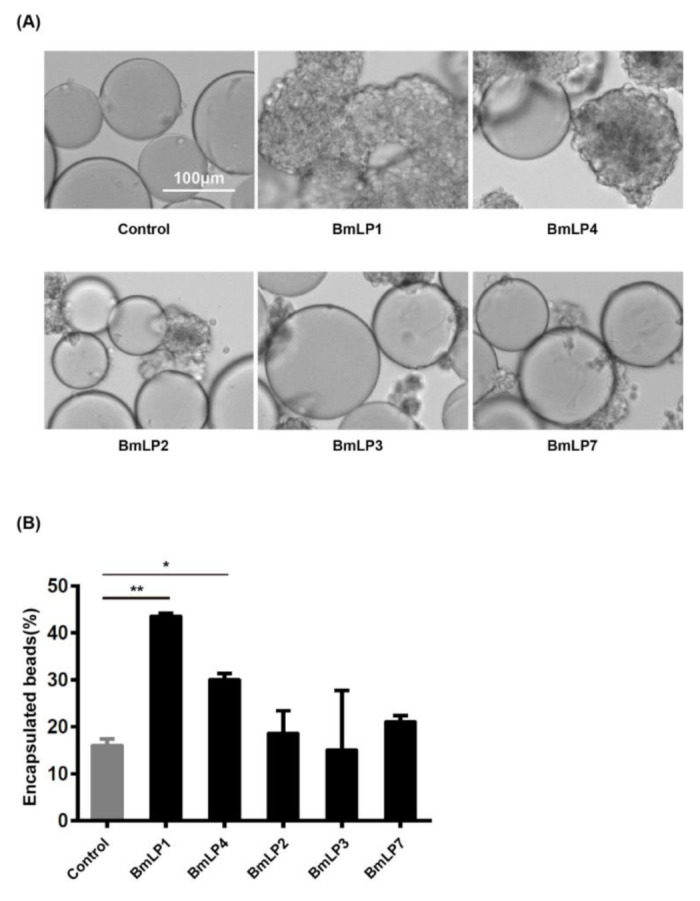
Encapsulation was performed using BmLP1, BmLP2, BmLP3, BmLP4, BmLP7 and no protein-coated beads (**A**). Ni-NTA agarose beads coated with recombinant BmLP1, BmLP2, BmLP3, BmLP4 and BmLP7 were incubated with hemocytes. Encapsulation of the protein-coated beads were observed by microscopy after 6 h of incubation. No protein-coated beads were used as the control. The numbers of encapsulated beads were computed (**B**). The vertical bars represent the mean ± SE (n = 2). Student’s *t*-test was used to calculate statistical significance. ** *p* < 0.01, * *p* < 0.1 versus the control. The error bars indicate the standard error of the mean (n = 2).

**Figure 7:**
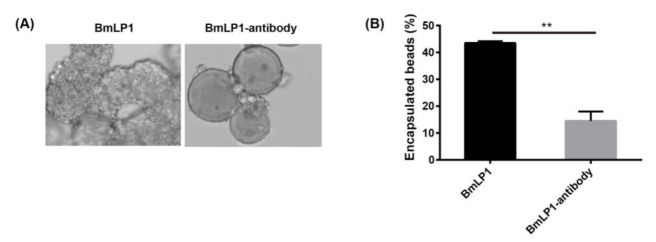
The encapsulation of BmLP1 was verified using BmLP1 specific polyclonal antibodies (**A**). BmLP1-coated beads were preincubated with BmLP1 specific polyclonal antibodies. Antibody-bound beads were incubated with hemocytes. Encapsulation of the antibody-bound beads was observed by microscopy after 6 h. The numbers of encapsulated beads were computed (**B**). The vertical bars represent the mean ± SE (n = 2). Student’s *t*-test was used to calculate statistical significance. ** *p* < 0.01 versus the control. The error bars indicate the standard error of the mean (n = 2).

These results suggest that BmLP1 and BmLP4 might recruit hemocytes and promote hemocyte encapsulation. To investigate whether the recombinant BmLP1 and BmLP4 can enhance cellular encapsulation, an in vitro encapsulation assay using protein-coated agarose beads was performed. The results showed that the beads coated with recombinant BmLP1 and BmLP4 were encapsulated by hemocytes within 6 h of incubation (Figure 6A,B). BmLP1 specific polyclonal antibody-bound beads effectively blocked hemocyte encapsulation within 6 h of incubation (Figure 7A). Approximately 43.5% of the beads coated with recombinant BmLP1, but only 14.5% of the BmLP1 specific polyclonal antibody-bound beads, were encapsulated (Figure 7B). These results demonstrate that the recombinant BmLP1 can recruit hemocytes and promote hemocyte encapsulation.

## 4. Discussion

Components of the silkworm hemolymph include 30K proteins, and previous reports have stated that BmLP1 can bind to *β*-1,3 glucan and interfere with fungal growth [21,22]. In the present study, we evaluated the defense mechanism of 30K proteins against fungi.

In the present study, the mRNA expression of 30K proteins was significantly upregulated after the injection of *β*-1,3 glucan and mannan. The protein level was consistent with the mRNA level, suggesting that 30K proteins are involved in the immune response. We speculated that the two PAMPs could activate the same signaling pathway and induce 30K protein expression. As *β*-1,3 glucan and mannan are fungal PAMPs, 30K proteins may also be involved in the anti-fungal immune response.

The recognition of PAMPs by pattern-recognition receptors is the first step of defense against pathogen infection [28]. A previous study showed that BmLP1 can bind to *β*-1,3 glucan [22], suggesting that BmLP1 mediates the immune response through this coupling on the fungal cell wall. However, in the present study, we found that BmLP1 has only a weak ability to directly inhibit fungal growth (Supplementary Figure S2), suggesting that BmLP1 might be an immune-recognition molecule, and not an immune effector.

Lectins, as immune-recognition molecules, have been found to mediate cellular immune responses [29,30]. The typical 30K CTD is similar to a lectin [19], and it can bind to PAMPs [22]. In the present study, we used different 30K proteins, as controls, in combination, and found that BmLP1, BmLP4, and BmLP7 can bind to fungi (*C. albicans*), owing to the ability of 30K proteins to bind to *β*-1,3 glucan. We speculate that 30K proteins may be involved in cell-mediated anti-fungal immune responses, similar to lectins.

Lectins bind not only to PAMPs, but also to hemocytes, and trigger cellular immunity [29,31], including pattern recognition, nodule formation, encapsulation, melanization, phagocytosis, and proPO activation [32,33,34]. The pathogens were encapsulated by multiple layers of hemocytes [27,29]. In the present study, we found that BmLP1 and BmLP4 can bind to the hemocyte cell membrane and promote encapsulation; however, they cannot bind to HeLa cells (Supplementary Figure S3). Furthermore, we found that the BmLP1 specific polyclonal antibodies can block hemocyte encapsulation. These results suggest that 30K proteins play a critical role in cellular immunity in the silkworm, and we speculate that 30K proteins activate cellular immunity by binding to the receptors on the hemocyte membrane.

It has been reported that BmLP3, unlike BmLP1, cannot bind to the cell membrane [35]. In the present study, BmLP1 and BmLP4 were detected in the cell membrane of hemocytes, whereas BmLP2, BmLP3, and BmLP7 penetrated into the cell, which is consistent with previous reports [35,36]. In 2012, Park et al., reported that the 30K protein BmLP3 (30Kc19) was a cell-penetrating protein, and in 2014, Park et al. identified a cell-penetrating peptide (VVNKLIRNNKMNC, Pep-c19) derived from BmLP3 by computational analysis of the primary structure, second structure, positive surface charge, hydrophobicity, and surface accessibility, which can efficiently penetrate cells. Multiple sequence alignment analysis showed that BmLP7 had the same polypeptide as BmLP3, but BmLP1, BmLP2, and BmLP4 did not have similar polypeptides. Since BmLP2, BmLP3, and BmLP7 were found to penetrate cells efficiently, we speculated that Bmlp2 might have different cell-penetrating peptides than BmLP3 and BmLP7. BmLP1 and BmLP4 may not contain these cell-penetrating peptides, and therefore they bind to the cell membrane but do not enter the cell. The dimerization of peptides or proteins is important for them to penetrate cells [37,38]. The dimerization of BmLP3 has previously been reported [36], and BmLP2 and BmLP7 were shown to dimerize in this study (Figure 5C), whereas BmLP1 and BmLP4 did not (Figure 5C). A dimeric form of a portion of the typical 30K proteins was detected in our study; however, other typical 30K proteins could not dimerize. Therefore, a portion of the typical 30K proteins might mediate cellular immunity, which binds to the hemocyte membrane and cannot dimerize. Other proteins with cell-penetrating ability might have other functions.

## 5. Conclusions

Overall, we investigated the expression of 30K after challenging the silkworms with fungal PAMPs on day 1 of the fifth instar larval stage, and identified BmLP1 and BmLP4 as key molecules in the response to fungal infection in silkworms. We demonstrated that BmLP1 and BmLP4 recruit and mediate hemocytes to bind with fungi. Finally, the fungi were encapsulated by hemocytes; however, the mechanism by which BmLP1 and BmLP4 bind to hemocytes is still unknown, which should be clarified by future research. The present study provides insights into the 30K-mediated cellular immunity in silkworm.

## Supplementary Materials

The following are available online at https://www.mdpi.com/2075-4450/12/2/107/s1, Figure S1: The SDS-PAGE of recombinant 30K proteins after purified by NTA agarose affinity chromatography, Figure S2: Antifungal activity of BmLP1, Figure S3: Binding of BmLP1 to Hela cells, Table S1: Primers used in this study.
