# Supplementary material for: Five Silkworm 30K Proteins Are Involved in the Cellular Immunity against Fungi
Source: Insects. 2021 Jan 27;12(2):107. doi: 10.3390/insects12020107 (PMC7911669; doi:10.3390/insects12020107)
Supplement: Supplementary file 1 [file insects-12-00107-s001.pdf]

Figure S1

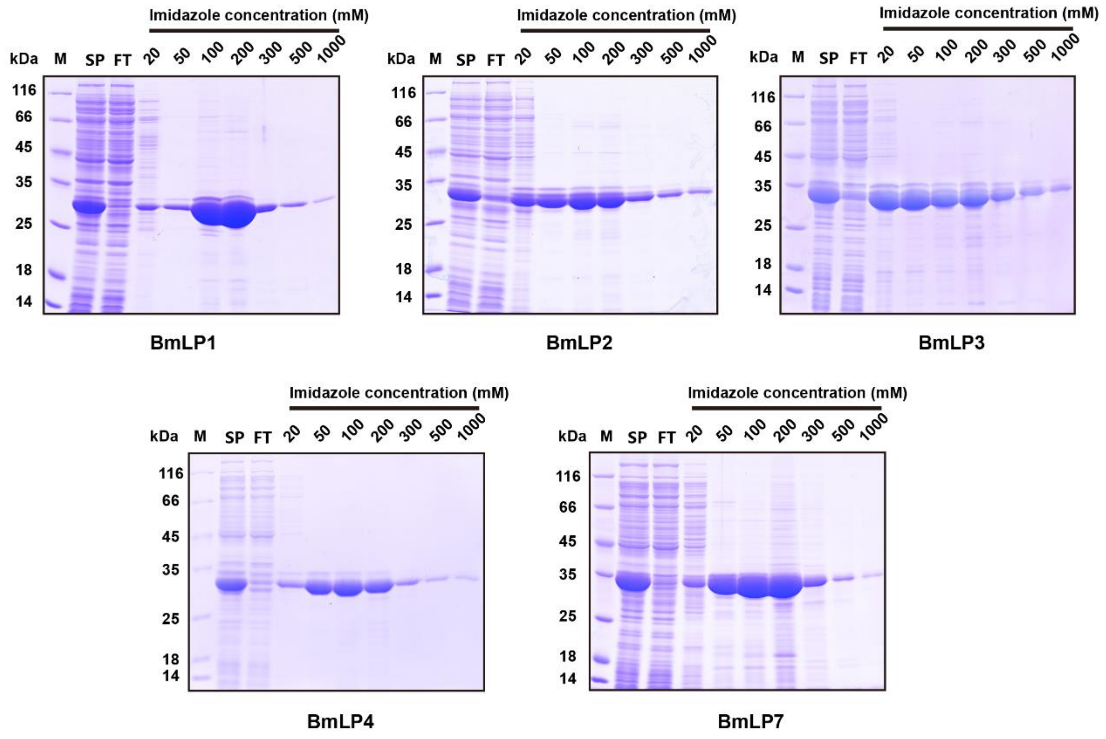

Figure S1. Recombinant 30K proteins were purified by NTA agarose affinity chromatography with a stepwise imidazole gradient. M: molecular weight marker; SP: soluble protein; FT: flow-through fraction.

Figure S2

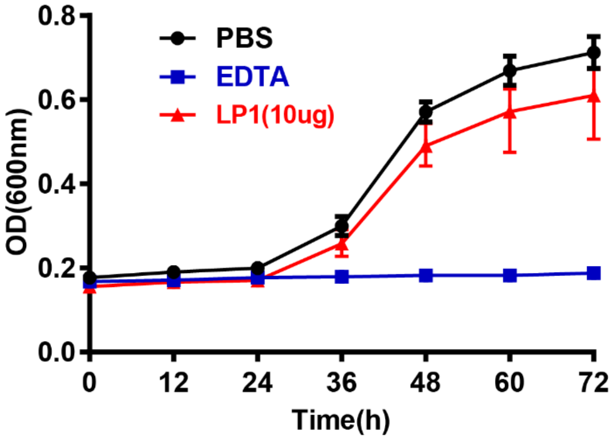

Figure S2. Antifungal activity of BmLP1 was performed by detecting the growth curve. 96-well plates were filled with 300μL of potato liquid medium containing 10μg BmLP1 and Candida albicans at a final concentration of 1 × 105 cells. 96-well plates were incubated at 30 °C, and fungal growth was observed

by monitoring the absorbance at 600 nm after culturing for 0, 12, 24, 36, and 48 h. Vertical bars represent the mean ± SE (n = 3).

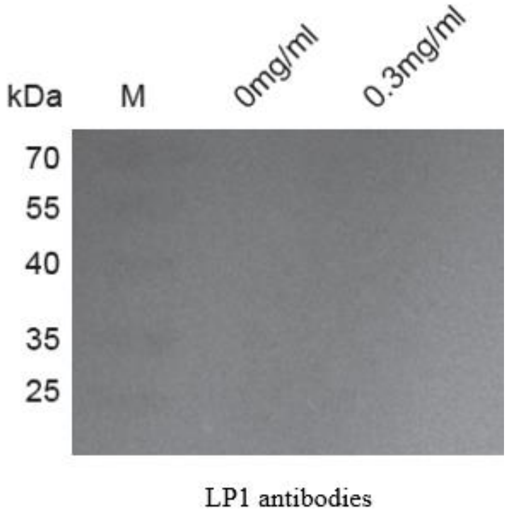

Figure S3. Binding of BmLP1 to Hela cells revealed using western blotting. 0.3mg/ml concentrations of purified proteins in DMEM medium were incubated with Hela cells for 2 h at room temperature, washed four times with PBS after trypsin digestion, and Hela cell proteins were extracted using RIPA. BmLP1 was detected using BmLP1 specific polyclonal antibodies.

Table S1. Primers used in this study. F: Forward primer; R: Reverse primer; The BmlpX-F/R for protein expression; The BmlpX qPCR-F/R for RT-qPCR.

Primers	Sequence (5'-3')
***Bmlp1*-F**	TACTTCCAATCCATGACACTTGCACCAAGAACTGA
***Bmlp1*-R**	TATCCACCTTTACTGTTAGTAGGGGACGATGTACC
***Bmlp2*-F**	TACTTCCAATCCATGGGCGTCGTGGAACTATCC
***Bmlp2*-R**	TATCCACCTTTACTGTTAGAAAGGTGTAATGAACC
***Bmlp3*-F**	TACTTCCAATCCATGGACGTCCCTAACGACATTCT
***Bmlp3*-R**	TATCCACCTTTACTGTTAGAAAGCCTTTATACCCC
***Bmlp4*-F**	TACTTCCAATCCATGGGCGTCACTGAAATGTCCG
***Bmlp4*-R**	TATCCACCTTTACTGCTAGAAAGGTGTAACGAACC
***Bmlp7*-F**	TACTTCCAATCCATGGCAGATTCCGACGTCCCT
***Bmlp7*-R**	TATCCACCTTTACTGTTAAAATGCCTTAACACCCC
***Bmlp1* qPCR-F**	TGTTATCGAGCTTCAGGT
***Bmlp1* qPCR-R**	CCCCATCCAGTTTAGAG
***Bmlp2* qPCR-F**	AGCACCAGGGAGCAAT
***Bmlp2* qPCR-R**	TCACCGTCGTGTCCAA
***Bmlp3* qPCR-F**	GAGGAGAAGAAGAGCGAAGT
***Bmlp3* qPCR-R**	GCCCTGGAGCCAAAGT
***Bmlp4* qPCR-F**	ACACCACCAGGGAGCA
***Bmlp4* qPCR-R**	TGTAACGAACCAGGAGAAG
***Bmlp7* qPCR-F**	GGAGAAGAAGAGCGAAGT
***Bmlp7* qPCR-R**	TGGAGCCAAAGTTGATAG
